# Evaluation of under-testing and under-diagnosis of tick-borne encephalitis in Germany

**DOI:** 10.1186/s12879-023-08101-6

**Published:** 2023-03-07

**Authors:** Katharina Schley, Josephine Friedrich, Andreas Pilz, Liping Huang, Bridget L. Balkaran, Martine C. Maculaitis, Claudius Malerczyk

**Affiliations:** 1 Pfizer Pharma GmbH, Linkstr. 10, 17085 Berlin, Germany; 2 Pfizer Corporation, Austria Gesellschaft M.B.H., Floridsdorfer Hauptstraße 1, 1210 Vienna, Austria; 3 Pfizer Inc., 235 East 42Nd Street, New York, NY USA; 4 Cerner Enviza, 51 Valley Stream Parkway, Malvern, PA USA

**Keywords:** Central nervous system, Encephalitis, Germany, Meningitis, Myelitis, Tick, Tick bite, Tick borne encephalitis, Viral testing

## Abstract

**Background:**

Tick-borne encephalitis (TBE), a viral infectious disease affecting the central nervous system, potentially resulting in prolonged neurological symptoms and other long-term sequelae. Case identification can be challenging as TBE can be associated with non-specific symptoms, and even in cases consistent with typical TBE symptoms, the rate of laboratory testing to confirm cases is unknown. This study assessed real-world TBE laboratory testing rates across Germany.

**Methods:**

In this retrospective cross-sectional study, physicians provided data on TBE decision-making, laboratory testing (serological), and diagnostics behavior via in-depth qualitative interviews (N = 12) or a web-based quantitative survey of their patient medical records (N = 166). Hospital-based physicians who specialized in infectious disease, intensive care unit, emergency room, neurology, or pediatrics with experience managing and ordering testing for patients with meningitis, encephalitis, or non-specific central nervous system symptoms in the past 12 months were included. Data were summarized via descriptive statistics. TBE testing and positivity rates were assessed for the aggregate sample of 1400 patient charts and reported by presenting symptoms, region, and tick bite exposure.

**Results:**

TBE testing rates ranged from 54.0% (non-specific neurological symptoms only) to 65.6% (encephalitis symptoms only); the percentage of TBE positive results ranged from 5.3% (non-specific neurological symptoms only) to 36.9% (meningitis symptoms only). TBE testing rates were higher among those with a tick bite history and/or who presented with headache, high fever, or flu-like symptoms.

**Conclusions:**

The findings of this study suggest that patients with typical TBE symptoms are likely under-tested, thus likely leading to under-diagnosis in Germany. To ensure appropriate case identification, TBE testing should be consistently integrated into routine practice for all patients who present with relevant symptoms or exposure to common risk factors.

**Supplementary Information:**

The online version contains supplementary material available at 10.1186/s12879-023-08101-6.

## Background

Tick-borne encephalitis (TBE) is a viral infectious disease that can affect the central nervous system (CNS) and can result in long-term neurological symptoms and even death [1]. TBE is endemic in Asia and in central, eastern, and northern Europe, with 10,000–15,000 cases each year [2]. In 2020, 3817 TBE cases were reported from 24 European Union (EU)/European Economic Area (EEA) countries [3]. In disease-endemic areas, people doing outdoor leisure activities (e.g., dog walking, gardening, jogging) and with recreational or occupational exposure to rural or outdoor settings (e.g., hunters, campers, forest workers, farmers) are potentially at risk for infection after a bite from a tick infected by the TBE virus. Furthermore, as mobility increases and tourism expands, travel to areas where the TBE virus circulates will broaden the population at risk for TBE infection [4]. In 2012, the European Centre for Disease Prevention and Control (ECDC) included TBE in the list of notifiable diseases in the EU [5], although TBE has already been a notifiable disease in Germany since 2001 [6].

TBE is a major public health concern due to potentially severe health outcomes caused by the viral infection. Furthermore, currently, no causal therapy against TBE exists, and patients can only be treated symptomatically [7]. Initial TBE symptoms may include fever, malaise, anorexia, muscle aches, headache, nausea, and/or vomiting. After a temporary disappearance of these symptoms, a recurrence of fever a few days later marks the beginning of the second phase of the disease. These manifestations involve the CNS, with symptoms such as meningitis, meningoencephalitis, or myelitis [7, 8]. Moreover, the typical symptoms of TBE encephalitis, which can be long-lasting, include ataxia and other potential complications, such as paralysis, headache, tiredness, and difficulties with concentration and memory; these symptoms and their potential for long-term impact on patient health further underline the severity of TBE and need for prevention, for which proper diagnosis is a prerequisite to ensure accurate epidemiology and case detection. However, diagnosing TBE is difficult due to often non-specific symptoms [9].

Findings from standard laboratory blood tests for patients presenting with non-specific symptoms during the first phase of TBE, such as leukopenia, thrombocytopenia or liver function tests, are not indicative of a TBE infection and therefore, an additional TBE-specific test is necessary to confirm a TBE case [8]. Although blood tests performed after the onset of neurological disease during the second phase of TBE may show an increase in the number of white blood cells in the blood and the cerebrospinal fluid (CSF), these findings still need to be confirmed with TBE-specific laboratory testing [8]. The TBE virus can be isolated from the blood during the first phase of the disease; the Guideline on TBE issued by the German Society of Neurology mentions polymerase chain reaction (PCR) as a method of diagnosing TBE infection, however, stating “TBE RNA detection in cerebrospinal fluid by PCR is usually only useful in the early phase of the disease (most likely in the prodromal phase), when no antibodies are detectable and pleocytosis in the cerebrospinal fluid is still missing” [10]. In clinical practice, laboratory diagnosis usually depends on the detection of specific immunoglobulin M (IgM) in either blood or CSF during the second phase of the disease [5, 7].

Manifestations, such as meningitis, encephalitis, meningoencephalitis, or meningoencephalomyelitis, often require hospitalization and supportive care based on syndrome severity [7]. Vaccination to prevent TBE has been available for > 40 years and is proven effective at preventing disease and potential long-term serious health consequences; however, prior research has found that vaccination uptake is low even in TBE-endemic areas in Germany, with declines observed in rates of compliance with subsequent vaccinations for TBE [11]. Schley et al. found that the yearly initiation rate of a primary immunization ranged between < 1% and 3% on federal state level [11]. Health claims analysis by the Robert-Koch Institute showed that the vaccination uptake ranged from 9.7% to 29.3%, on average, per federal state within risk areas [12]. Most recently, a study by Ghiani et al. showed that the identification of new risk areas substantially increases vaccination uptake in those newly designated risk areas [13].

In Germany, most districts in Bavaria and Baden-Wuerttemberg, southern Hesse, southeastern Thuringia, and Saxony are designated as TBE risk areas [14, 15]. Central Hesse, Saarland, Rhineland-Palatinate, and Lower Saxony comprise individual risk areas, with five new districts in Bavaria, Hesse, Saxony, Thuringia, and Saxony-Anhalt designated as risk areas in 2021 [14]. In 2022, six new risk areas in Brandenburg, North Rhine-Westphalia, and Saxony were added, bringing the total to 175 districts in Germany currently designated as TBE risk areas [15]. Recent research has suggested that knowledge of TBE disease and risks may be inadequate among some physicians, especially in areas currently defined as non-risk areas, which could potentially contribute to suboptimal vaccination and case identification rates [16]. Inadequate TBE testing and diagnosis impacts TBE surveillance, which is interlinked with low awareness and under-testing, leading to a vicious cycle of continued under-ascertainment of TBE cases [17]. The societal burden of TBE under-diagnosis likely has long-term consequences for individuals, healthcare providers, and payers, particularly in endemic areas [17].

A better understanding of the proportion of cases with TBE-consistent symptoms that are not tested for TBE is vital to inform clinical decision-making, as well as to raise greater awareness about TBE disease and risks among physicians and the general public, alike. Accordingly, this study aimed to understand the TBE testing and diagnostic pathway and to estimate potential TBE under-testing among patients presenting with TBE-consistent symptoms in Germany.

## Methods

### Study design

This retrospective cross-sectional study was conducted in Germany among hospital-based physicians. The study was granted an exemption determination from a central Institutional Review Board (IRB) in the United States prior to starting data collection. No personal identifiable information was captured during the course of the study. Prior to participating, physicians provided their informed consent to proceed with the study.

**Fig. 1:**
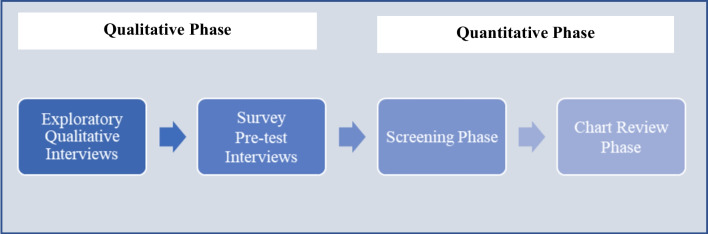
Study schematic

The study was conducted in two main phases (Fig. 1). A qualitative phase was initially conducted between July 13, 2020 and August 13, 2020 in which 12 physicians were interviewed to assess how TBE is diagnosed and managed in real-world practice, as well as to examine the feasibility of questions to be included in the quantitative phase. The quantitative phase, which was conducted between October 14, 2020 and May 7, 2021, consisted of two parts, a screening and a chart review survey.

To be eligible to participate in either phase of the study, physicians must have reported (1) being, as their primary specialty, an emergency room (ER) specialist, an intensive care unit (ICU) physician (i.e., medical, neurological, or pediatric ICU), an infectious disease specialist, a neurologist, or a pediatrician, (2) being in clinical practice for ≥ 3 years, and (3) spending ≥ 60% of their time in clinical practice. Qualitative phase participants must have also reported (1) working in a hospital-based setting ≥ 70% of the time and (2) managing ≥ 2 patients with meningitis, encephalitis, or non-specific CNS symptoms per year and prescribing or ordering testing for some patients. Quantitative phase participants must have also reported (1) working in a hospital-based setting ≥ 50% of the time and (2) managing ≥ 5 patients with meningitis, encephalitis, or non-specific CNS symptoms per year and prescribing or ordering testing for these patients.

In the quantitative phase, physicians (N = 500) were first screened in order to identify up to 200 who met the eligibility criteria for the chart review survey, with an approximately even split of ER specialists, ICU physicians, infectious disease specialists, neurologists, and pediatricians/neuro-pediatricians, and to gauge the caseload of patients with meningitis, encephalitis, or myelitis symptoms among the physician specialties of interest.

Prior to chart review survey data collection, the survey instrument was piloted with a convenience sample of 5 physicians who met the eligibility criteria described above to verify that the questions were appropriate and sufficiently clear to respondents and that the required data points were easy to collect (to reduce the amount of potential missing data). For the chart review, physicians completed a 50-min cross-sectional web-based survey, including a minimum of 2–3 retrospective case report forms (CRFs) for patients who had presented with meningitis and those who presented with encephalitis. The chart review survey collected profile information about the physician (e.g., gender, age, specialty, practice setting, etc.) and about their clinical practice (e.g., number of patients with meningitis, encephalitis, and myelitis seen in the past year with etiology, diagnostic testing performed, etc.).

### Data analysis

For the qualitative interviews, results were summarized with means (continuous data) or counts and percentages (categorical data), as well as with illustrative verbatim quotes. For the chart review survey, sample characteristics variables were reported as counts and percentages. The count and percentage of patients who received a TBE test and who did not receive a TBE test were also reported. The TBE positive rate was computed as the number of patients who had a positive TBE test divided by the number of patients who received a TBE test and then multiplied by 100 to convert to a percentage value. TBE testing rate and TBE positive rate were computed among the subsets of patients who experienced different types of symptoms for the aggregate patient sample, tick bite (tick bite, no tick bite, and don’t know), seasonality (admitted during tick season^1^and not admitted during tick season), headache (headache and no headache), fever groups (no fever, fever > 38 °C to 39 °C, and high fever > 39 °C), clinical manifestations and flu-like symptoms (yes or no). Chi-square tests were used to examine whether the distribution of TBE positive rate varied across the five manifestations of interest (i.e., meningitis only, encephalitis only, myelitis only, a combination of meningitis, encephalitis, and/or myelitis, and non-specific neurological symptoms) for the aggregate sample, for patients who presented with headache, and for those who presented without headache. p-values < 0.05, two-tailed, were considered statistically significant.

## Results

### Qualitative interviews

#### Physician characteristics

**Fig. 2:**
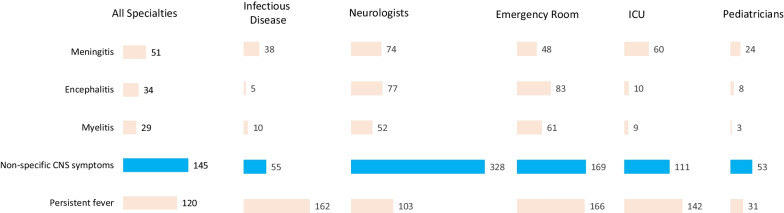
Mean number of patients seen each year by physician specialty and symptom type. *CNS* central nervous system, *ICU* intensive care unit

The 12 physicians who participated in the qualitative interviews reported a mean of 16.6 (range: 7–23) years in practice. Half were either neurologists or ICU physicians (n = 6, 50.0%), and most (n = 10, 83.3%) were in practice in endemic regions. Three-quarters of physicians were in practice at either a university hospital (n = 5, 41.7%) or at a public hospital (n = 4, 33.3%). As shown in Fig. 2, across all specialties, the highest mean number of patients seen annually by physicians presented with non-specific CNS symptoms (145.0), followed by persistent fever (120.0).

*“The majority of patients who come have non-specific CNS symptoms such as headaches, nausea, fatigue, sometimes vision problems, seizures.” (Pediatrician/Endemic Region)*

*“The [patients] mostly come with unspecific CNS symptoms.” (ER Specialist/Non-endemic Region)*

#### Role in testing

According to physicians’ interview responses, ER physicians are usually the first contact that patients have within the hospital; thus, they are, in most cases, the primary specialist who will obtain the patient's medical history and carry out the first appropriate diagnostic tests. The ER specialist will initiate empirical therapy in acute cases and may refer the patient to other wards for further diagnostics/treatment. Neurologists will see patients on referral from the ER or are called to the ER for diagnosis. Neurologists may also take over the diagnosis completely if there is a suspicion of a neurological disease (e.g., meningitis) immediately upon admission of the patient. Based on the results of the qualitative analysis, laboratory analyses, a lumbar puncture, and a computed tomography (CT) scan may be ordered. The participants stated that there is no clear time when ICU specialists are called in. In some cases, they are already called in at the ER, where they are mainly involved in their function as internists. For pediatricians, it depends on which wards/areas of the clinic they work, with the procedure within the clinic being similar to that for adult patients. Infectious disease specialists are only consulted at a later stage.

#### Testing and diagnostic pathway

As noted by physicians in their interview responses, there are some common testing and diagnostic procedures across hospitals; however, each hospital and each department can develop its own standard diagnostic procedures. Nearly all physicians reported that the following tests were always ordered, with the usual diagnostic pathway consisting of: (1) obtaining the patient’s medical history and anamnesis (especially in case of non-specific CNS symptoms), (2) ordering a blood test, and (3) performing a lumbar puncture to examine CSF. For blood tests, inflammation parameters, kidney values, and liver values are assessed. In this step, the physician will initially try to distinguish whether it is infectious or not, and in the latter case, whether it is a bacterial or viral inflammation. If further investigation is needed, imaging examinations, such as mediator release test and/or CT and, in rare cases, an electroencephalogram or a panel of infectious disease PCR tests, are ordered per the clinical pathway recommended by the German Association of the Scientific Medical Societies [19].

*“By default, you would draw blood, do the imaging, from the head the imaging is first, and then the lumbar puncture. Usually, you do the imaging first, and then the lumbar puncture if circumstances allow.” (Neurologist/Endemic Region).*

*“The standard procedure is that you try to make a pathogen detection from blood and CSF”. (Infectious disease Specialist/Endemic Region)*

*“First, we differentiate between non-specific, encephalitis, or myelitis. Then, whether it is infectious or not. Then, the next thought is to lose as little time as possible and to run fast and sound infectiological diagnostics." (Infectious Disease Specialist/Non-endemic Region).*

*“We have an SOP [standard operating procedure], but I think every practitioner does it differently.” (Infectious Disease Specialist/Non-endemic Region)*

Physicians were asked to rate various factors that may impact the testing and diagnostic pathway. Only clinical symptoms and patient history were perceived to have a high/very high impact on testing and diagnostic pathway, with nearly all other factors, including availability and cost of a test, viewed by physicians as having low/very low impact (Table 1).

**Table 1:** Perceived Importance of Factors Affecting Testing or Diagnosis

Criteria	How can it impact the diagnostic process?	Perceived impact
Clinical presentations	The decision to run some specific tests is always driven by the clinical symptoms	1
Patients medical, geographic, or past activities history	Physicians mentioned that travel activity or being outdoors has some influence	2
Previous laboratory tests	Most relevant tests are carried out within the clinic. They usually do not re-do a test ordered by another department, except if there is a good reason	3
Time of a year (seasonality)	The seasons in and of themselves have no influence on the test. However, some doctors are more sensitive to TBE; all doctors tend to think of TBE in the warmer seasons when arthropods, such as ticks, are more active, and people are outdoors more often	4
Availability of a test	Availability only plays a minor role, as physicians always have access to an external laboratory, if necessary	4
Costs of tests	Cost also plays a minor role; tests are usually reimbursed. A few mentioned that they do not always order a PCR test because of the cost	4
How soon to receive the result?	Physicians receive most of the test results within 1 day. However, ELISA tests can take up to 4 days	4
Reliability (sensitivity and specificity) of a test	This is not a concern	5
Convenience or access of a test	This is not a concern	5

Each criterion was rated on a scale from 1 (very high impact) to 5 (very low impact). ELISA: enzyme-linked immunoassay; PCR: polymerase chain reaction; TBE: tick-borne encephalitis

### Chart review survey

#### Sample characteristics

Overall, 166 physicians collectively provided 1,400 patient charts, which were included in the final quantitative analyses. Most physicians (60.8%) worked in hospitals in a non-endemic region, followed by endemic (19.9%), middle endemic (13.3%), and low endemic (6.0%) regions.^2^ Roughly half of the patients were male (50.9%) and had been admitted to the hospital during tick season (55.4%); most were adults aged > 20 years (81.6%) and resided in a non-endemic region (61.5%). Patients most often presented with meningitis (33.9%) and least often presented with non-specific neurological symptoms (25.1%). The most frequently reported TBE risk factors among patients were outdoor activities in forests or grassy areas (39.9%), travelling (22.9%), and tick bite (22.1%).

#### TBE testing and positive rates

A majority (60.6%) in the sample of 1400 patients were tested for TBE (Table 2). The TBE testing rate was highest among patients presenting with encephalitis only (65.6%) and lowest among those presenting with non-specific neurological symptoms (54.0%). TBE positive rate was highest among patients presenting with meningitis only (36.9%). Those presenting with non-specific neurological symptoms showed the lowest positive rate of 5.3%, followed by patients presenting with myelitis only (7.4%). The TBE positive rate distribution varied across the different categories of presenting symptoms (i.e., meningitis only, encephalitis only, myelitis only, a combination of meningitis, encephalitis, and/or myelitis, and non-specific neurological symptoms; p < 0.001). More than one-third of patients with very typical TBE symptoms, like meningitis (37.9%), encephalitis (34.4%), or a combination of meningitis, encephalitis and/or myelitis (37.3%), were not tested (Table 2).

**Table 2:** TBE under-testing and under-diagnosis: aggregate sample

Patients	N	TBE tested, n (%)	Not TBE tested, n (%)	TBE positive, n (%)	TBE positive, p-value
All patients	1400	848 (60.6)	552 (39.4)	169 (19.9)	NA
Meningitis Only	401	249 (62.1)	152 (37.9)	92 (36.9)	< 0.001
Encephalitis Only	349	229 (65.6)	120 (34.4)	41 (17.9)	
Myelitis Only	232	135 (58.2)	97 (41.8)	10 (7.4)	
Combination of Meningitis, Encephalitis, and/or Myelitis	1049	658 (62.7)	391 (37.3)	157 (23.9)	
Non-specific Neurological Symptoms (exclusive)	313	169 (54.0)	144 (46.0)	9 (5.3)	

Percentages values are row percentages. NA: not applicable; TBE: tick-borne encephalitis

Nearly three-quarters (72.8%) of all patients presenting with tick bite were tested for TBE, whereas a little over half of patients with no tick bite (57.6%) or “don’t know” (55.4%) were tested (Table 3). Among patients presenting with tick bite, the highest TBE positive rates were observed for those presenting with meningitis only (74.4%), followed by those presenting with a combination of meningitis, encephalitis, and/or myelitis symptoms (72.7%). TBE positive rates ranged from 2.3% (non-specific neurological symptoms) to 17.9% (combination of meningitis, encephalitis, and/or myelitis symptoms) for patients with no tick bite and from 6.7% (non-specific neurological symptoms) to 16.7% (a combination of meningitis, encephalitis, and/or myelitis symptoms) for those patients with “don’t know” for history of tick bite (Table 3).

**Table 3:** TBE under-testing and under-diagnosis by tick bite status

Patients	N	TBE tested, n (%)	Not TBE tested, n (%)	TBE positive, n (%)
Tick bite				
All patients	298	217 (72.8)	81 (27.2)	119 (54.8)
Meningitis Only	116	90 (77.6)	26 (22.4)	67 (74.4)
Encephalitis Only	80	63 (78.8)	17 (21.3)	29 (46.0)
Myelitis Only	50	27 (54.0)	23 (46.0)	10 (37.0)
Combination of Meningitis, Encephalitis, and/or Myelitis	12	11 (91.7)	1 (8.3)	8 (72.7)
Non-specific Neurological Symptoms (exclusive)	40	26 (65.0)	14 (35.0)	5 (19.2)
No tick bite				
All patients	979	564 (57.6)	415 (42.4)	43 (7.6)
Meningitis Only	264	147 (55.7)	117 (44.3)	24 (16.3)
Encephalitis Only	246	156 (63.4)	90 (36.6)	11 (7.1)
Myelitis Only	175	105 (60.0)	70 (40.0)	NA
Combination of Meningitis, Encephalitis, and/or Myelitis	48	28 (58.3)	20 (41.7)	5 (17.9)
Non-specific Neurological Symptoms (exclusive)	246	128 (52.0)	118 (48.0)	3 (2.3)
Don’t know				
All patients	83	46 (55.4)	37 (44.6)	4 (8.7)
Meningitis Only	21	12 (57.1)	9 (42.9)	1 (8.3)
Encephalitis Only	21	10 (47.6)	11 (52.4)	1 (10.0)
Myelitis Only	7	3 (42.9)	4 (57.1)	NA
Combination of Meningitis, Encephalitis, and/or Myelitis	7	6 (85.7)	1 (14.3)	1 (16.7)
Non-specific Neurological Symptoms (exclusive)	27	15 (55.6)	12 (44.4)	1 (6.7)

Percentages values are row percentages. When the number of confirmed TBE cases is 0, there will not be estimates for TBE positive rate, as confirmed TBE cases are needed to estimate these values. NA: not applicable; TBE: tick-borne encephalitis

Patients presenting with high fever (71.9%) had a higher TBE testing rate than those presenting with fever (61.9%) and those with no fever (44.6%) (Table 4). Among patients presenting with high fever, the highest TBE positive rates were observed for those presenting with meningitis only (39.5%), followed by those presenting with a combination of meningitis, encephalitis, and/or myelitis symptoms (29.1%). Among patients presenting with fever, the highest TBE positive rates were found for those presenting with a combination of meningitis, encephalitis, and/or myelitis symptoms (19.0%) and those presenting with meningitis only (18.5%). TBE positive rates ranged from 4.8% (non-specific neurological symptoms) to 35.1% (meningitis only) for patients with no fever (Table 4).

**Table 4:** TBE under-testing and under-diagnosis by fever status

Patients	N	TBE tested, n (%)	Not TBE tested, n (%)	TBE positive, n (%)
High fever (> 39 °C)				
All patients	540	388 (71.9%)	152 (28.1%)	97 (25.0%)
Meningitis Only	232	157 (67.7%)	75 (32.3%)	62 (39.5%)
Encephalitis Only	166	124 (74.7%)	42 (25.3%)	29 (23.4%)
Myelitis Only	83	65 (78.3%)	18 (21.7%)	10 (15.4%)
Combination of Meningitis, Encephalitis, and/or Myelitis	447	323 (72.3%)	124 (27.7%)	94 (29.1%)
Non-specific Neurological Symptoms (exclusive)	93	65 (69.9%)	28 (30.1%)	3 (4.6%)
Fever (> 38 °C to 39 °C)				
All patients	443	274 (61.9%)	169 (38.1%)	46 (16.8%)
Meningitis Only	264	173 (65.5%)	91 (34.5%)	32 (18.5%)
Encephalitis Only	154	96 (62.3%)	58 (37.7%)	16 (16.7%)
Myelitis Only	68	50 (73.5%)	18 (26.5%)	2 (4.0%)
Combination of Meningitis, Encephalitis, and/or Myelitis	342	211 (61.7%)	131 (38.3%)	40 (19.0%)
Non-specific Neurological Symptoms (exclusive)	101	63 (62.4%)	38 (37.6%)	6 (9.5%)
No fever				
All patients	417	186 (44.6%)	231 (55.4%)	26 (14.0%)
Meningitis Only	65	37 (56.9%)	28 (43.1%)	13 (35.1%)
Encephalitis Only	108	60 (55.6%)	48 (44.4%)	8 (13.3%)
Myelitis Only	105	37 (35.2%)	68 (64.8%)	2 (5.4%)
Combination of Meningitis, Encephalitis, and/or Myelitis	260	124 (47.7%)	136 (52.3%)	23 (18.5%)
Non-specific Neurological Symptoms (exclusive)	157	62 (39.5%)	95 (60.5%)	3 (4.8%)

Percentages values are row percentages. TBE: tick-borne encephalitis

Patients presenting with headache had a higher TBE testing rate than those with no headache (69.9% vs. 46.2%) (Table 5). Among patients presenting with headache, the highest TBE positive rates were observed for those presenting with meningitis only (34.8%), followed by those presenting with a combination of meningitis, encephalitis, and/or myelitis symptoms (25.6%). TBE positive rates ranged from 5.9% (myelitis only) to 41.5% (meningitis only) for patients with no headache. The distribution of TBE positive rate varied across the different categories of presenting symptoms (i.e., meningitis only, encephalitis only, myelitis only, combination of meningitis, encephalitis, and/or myelitis, and non-specific neurological symptoms) among those who presented with a headache and among those who presented without a headache (both, p < 0.001) (Table 5).

**Table 5:** TBE under-testing and under-diagnosis by headache status

Patients	N	TBE tested, n (%)	Not TBE tested, n (%)	TBE positive, n (%)	TBE positive, p-value
Headache					
All patients	855	597 (69.8%)	258 (30.2%)	128 (21.4%)	NA
Meningitis Only	342	230 (67.3%)	112 (32.7%)	80 (34.8%)	< 0.001
Encephalitis Only	279	207 (74.2%)	72 (25.8%)	43 (20.8%)	
Myelitis Only	108	84 (77.8%)	24 (22.2%)	10 (11.9%)	
Combination of Meningitis, Encephalitis, and/or Myelitis	660	472 (71.5%)	188 (28.5%)	121 (25.6%)	
Non-specific Neurological Symptoms (exclusive)	195	125 (64.1%)	70 (35.9%)	7 (5.6%)	
No headache					
All patients	543	251 (46.2%)	292 (53.8%)	41 (16.3%)	NA
Meningitis Only	133	65 (48.9%)	68 (51.1%)	27 (41.5%)	< 0.001
Encephalitis Only	147	73 (49.7%)	74 (50.3%)	10 (13.7%)	
Myelitis Only	148	68 (45.9%)	80 (54.1%)	4 (5.9%)	
Combination of Meningitis, Encephalitis, and/or Myelitis	387	186 (48.1%)	201 (51.9%)	36 (19.4%)	
Non-specific Neurological Symptoms (exclusive)	156	65 (41.7%)	91 (58.3%)	5 (7.7%)	

Percentages values are row percentages. NA: not applicable; TBE: tick-borne encephalitis

Similar to the presence of high fever, patients presenting with flu-like symptoms had a higher TBE testing rate than those with no flu-like symptoms (75.4% vs. 46.6%) (Table 6). Among patients presenting with flu-like symptoms, the highest TBE testing rates were observed for those presenting with encephalitis only (82.7%). Almost three-quarters of patients with flu-like symptoms presenting with either myelitis or meningitis, alone or in combination, and those with non-specific neurological symptoms were tested. TBE positive rate was highest for those presenting with myelitis and encephalitis among patients with flu-like symptoms.

**Table 6:** TBE under-testing and under-diagnosis by clinical manifestations and flu-like symptoms

Patients	N	TBE tested, n (%)	Not TBE tested, n (%)	TBE positive, n (%)
Flu-like symptoms				
All patients	1398	848 (60.7)	550 (39.3)	169 (19.9)
No	716	334 (46.6)	382 (53.4)	37 (11.1)
Yes	682	514 (75.4)	168 (24.6)	132 (25.7)
Clinical manifestations, flu like symptoms = yes				
All patients	678	513 (75.7)	165 (24.3)	132 (25.7)
Meningitis only	234	172 (73.5)	62 (26.5)	76 (44.2)
Encephalitis only	173	143 (82.7)	30 (17.3)	32 (22.4)
Myelitis only	105	79 (75.2)	26 (24.8)	8 (10.1)
NSNS only	120	89 (74.2)	31 (25.8)	7 (7.9)
Myelitis + encephalitis	4	2 (50.0)	2 (50.0)	2 (100.0)
Myelitis + meningitis	4	3 (75.0)	1 (25.0)	2 (66.7)
Encephalitis + meningitis	26	17 (65.4)	9 (34.6)	3 (17.6)
Encephalitis + NSNS	5	3 (60.0)	2 (40.0)	NA
Meningitis + NSNS	7	5 (71.4)	2 (28.6)	2 (40.0)
Clinical manifestations, flu like symptoms = no				
All patients	709	330 (46.5)	379 (53.5)	37 (11.2)
Meningitis only	167	77 (46.1)	90 (53.9)	16 (20.8)
Encephalitis only	174	86 (49.4)	88 (50.6)	9 (10.5)
Myelitis only	127	56 (44.1)	71 (55.9)	2 (3.6)
NSNS only	193	80 (41.5)	113 (58.5)	2 (2.5)
Myelitis + encephalitis	8	8 (100.0)	0 (0.0)	0 (0.0)
Myelitis + meningitis	0	NA	NA	NA
Encephalitis + meningitis	25	15 (60.0)	10 (40.0)	7 (46.7)
Encephalitis + NSNS	7	4 (57.1)	3 (42.9)	NA
Meningitis + NSNS	8	4 (50.0)	4 (50.0)	1 (25.0)

Percentages values are row percentages. When the number of confirmed TBE cases is 0, there will not be estimates for TBE positive rate, as confirmed TBE cases are needed to estimate these values. In the survey, physicians were asked to report the general signs and symptoms that the patient presented with and could select ≥ 1 of the following options: “bulging soft spot(s)”, “fever (> 38–39 °C)”, “high fever (> 39 °C)”, “flu-like symptoms”, “headache”, “mottled/blotchy skin”, “nausea/vomiting”, “pain”, “tachypnoea”, or “rash (skin/eyes/mouth)”. Data shown in the table reflects the selection of the “flu-like symptoms” response option and was not dependent upon or derived from the selection of any other general signs and symptoms that could be considered flu-like (e.g., headache or fever). NA: not applicable; NSNS: non-specific neurological symptoms; TBE: tick-borne encephalitis

Among patients admitted to the hospital during tick season, the TBE testing (75.6%) and TBE positive (47.6%) rates were highest for patients who presented with meningitis only (Additional file 1).

## Discussion

In the current study, we aimed to understand the decision tree for TBE testing and diagnosis and estimate under-testing and under-diagnosis of TBE cases in Germany. By integrating both qualitative and quantitative methodologies, this study provided novel insights that can serve to inform public health policy and clinical practice. Our findings suggest that critical gaps remain in the routine testing and diagnosis of TBE, despite growing awareness of TBE as a public health concern. For instance, the Task Force on the diagnosis and management of TBE of the European Academy of Neurology (EAN) defines patients with a history of exposure to ticks presenting with symptoms of meningitis, meningoencephalitis, encephalomyelitis, or myelitis as possible TBE cases [5], indicating that those patients should be tested for TBE. However, the results of this study revealed that more than one-third of patients with typical symptoms of TBE, such as meningitis and encephalitis either alone or in combination, were not tested, leading to an under-ascertainment of TBE cases.

In clinical practice, TBE testing and the correct identification of TBE cases might be unnecessary for the treatment of a TBE infection, as no causal treatment exists, and patients can only be treated symptomatically, which might contribute to under-testing of potential TBE cases. The accurate diagnosis of TBE is nevertheless necessary for several reasons, primarily to allow appropriate follow-up of patients for complete/incomplete recovery, to identify individuals and populations for future vaccination, and for surveillance of cases in which similar clinical findings are observed. However, insufficient awareness of the disease among physicians and a lack of routine screening outside of endemic regions may contribute to the missed diagnosis of TBE and has previously been reported as a causal factor in undiagnosed TBE cases in non-endemic regions [20]. Additionally, patients often do not recall the tick bite, which potentially further hampers considerations of a potential TBE infection, thus leading to under-testing and under-diagnosis. For instance, Nigrovic et al. found that only 18% of caregivers of children diagnosed with Lyme disease, a bacterial infection transmitted via ticks, recalled a preceding tick bite [21]. However, the correct identification of TBE cases is important to monitor the local epidemiological situation, to estimate individual risk, and to define new risk areas, as risk area designation in Germany is incidence-based. As such, under-testing can translate into the under-estimation of a notifiable disease, thus resulting in an under-estimation of the true disease burden/incidence, which can have policy implications, including the under-utilization of effective prevention measures. At the same time, the identification of risk areas is important to raise awareness of TBE disease and the associated individual risk, which might help to increase vaccination uptake. For instance, Ghiani et al. showed that vaccination uptake increases in newly designated risk areas in eastern federal states of Germany, highlighting the potential link between correct identification of cases, risk area designation, disease and risk awareness, and vaccination uptake [13].

As active immunization is the most effective way to protect against this possibly life-threatening disease, raising TBE awareness and vaccination uptake is urgently needed to decrease the burden of disease across Germany. Discussions are warranted to further encourage TBE vaccination among the general public, consistent with recommendations in international travel vaccination guidelines and checklists in Germany.

Our qualitative results indicate that TBE testing procedures are not standardized across hospitals in Germany, and variations sometimes may even exist in different departments within the same hospital. Given the observed variations in TBE testing procedures, it is possible that the extent of TBE under-testing and under-diagnosis that occurs may depend upon the specific hospital or department to which a patient is admitted. It will therefore be important to develop clear decision trees for TBE diagnostic testing, which can help to align testing processes across Germany.

### Limitations

Patient medical records may have missing information and errors or inconsistencies regarding diagnoses, tests, treatments, or other clinical variables. As is the case with all studies that rely on existing medical records, availability of information in records will vary by physician practice and will reflect differences in practice patterns, recording practices, and medical norms.

Data for this study were collected during the COVID-19 pandemic. As such, any accompanying strain on healthcare system resources may have influenced the patterns of TBE testing and case identification observed in the current study. A recent study assessing the impact of COVID-19 and associated pandemic-related public health restrictions on notifiable infectious diseases under surveillance in Germany showed that, irrespective of county population size or COVID-19 incidence rates, case numbers for all other notifiable infectious diseases decreased in 2020 among all age groups, relative to 2016–2019, except for TBE, which demonstrated a 58% increase in case numbers in 2020 over prior years [22]. It is possible that public health measures enacted during the COVID-19 pandemic, such as social distancing, travel limitations, and mask wearing, may have reduced transmission of most infectious diseases (i.e., fewer opportunities for person-to-person transmission). In contrast, pandemic-related social restrictions may have led individuals to greater engagement in outdoor recreational activities, which along with the high adult tick counts observed in 2020 in Germany [22, 23], could have increased potential exposure to ticks and subsequent risk of TBE, especially in endemic areas. A future study should evaluate longitudinal trends in under-testing to quantify the specific impact of the COVID-19 pandemic on rates of TBE testing and case identification.

Due to data protection, the analysis did not collect information on district level, the level at which risk areas are classified in Germany. The quantitative study sample was skewed toward physicians in non-endemic areas (Additional file 2), potentially due to the focus on particular practice specializations, rather than general practitioners. The small sample of endemic region physicians in this study may thus limit the ability to generalize the findings. Future research with larger samples is warranted to verify whether the current study’s results reflect TBE testing and diagnosis patterns in endemic regions and classified risk areas. Given the study focused on those physicians and specialties that most often test for and treat TBE, it is thus possible that the current study provides a very conservative estimate of under-testing for TBE and case identification. Also, physicians might recall their more recent or remarkable cases, which might influence the inclusion of patients and the results. Additional research is needed to determine the extent to which this study’s findings reflect the testing and diagnostic behaviors of the broader population of physicians and specialties across Germany.

## Conclusions

Findings suggest that patients with typical TBE symptoms are likely under-tested, indicating a potential under-diagnosis of TBE in Germany. To ensure appropriate case identification, TBE testing should be more consistently integrated into routine practice for all patients who present with relevant symptoms and those exposed to common risk factors, especially tick bite. An accurate understanding of risk areas, which are currently retrospectively defined based on the number of human incidences, is important to create awareness among the public and healthcare providers regarding TBE testing. Therefore, under-identification of TBE cases leads to an interlinked chain of events, with inadequate TBE testing and diagnosis impacting TBE surveillance efforts and potentially leading to a lack of TBE risk area identification, which then contributes to low awareness, continuous under-testing, and a low implementation of preventive measures, including vaccination. As part of a concerted public health strategy, it would be vital to increase vaccination rates and to highlight the awareness for travel vaccination within and to Europe and Germany, in particular, among the general public to protect against infection, given the lack of curative treatment for TBE, to ultimately reduce TBE case numbers and the associated disease burden. Future research needs to be done to further classify under-testing and under-diagnosis, for instance at the laboratory level, to understand more precisely how many samples are sent for TBE testing and to identify regional differences in TBE testing between endemic and non-endemic areas.

## Supplementary Information

**Additional file 1:** TBE Under-Testing and Under-Diagnosis by Seasonality; the table shows frequencies and percentages of patients tested for TBE, not tested for TBE, and TBE positive for the total sample and symptom types stratified by admitted during TBE season and not admitted during TBE season.

**Additional file 2:** Physicians Per Federal State: Observed Data Relative to the Population; the table shows the percentage of patients and the ratio of physicians per federal state observed in the current study to physicians per federal state in 2019 German population data for each of 16 federal states.

## Data Availability

The datasets generated and/or analyzed during the current study are not publicly available due to the data collection only being granted exemption determination from an IRB for this specific protocol, but they are available from the corresponding author upon reasonable request.

## Abbreviations

CNS: Central nervous system
COVID-19: Coronavirus disease 2019
CRF: Case report forms
CSF: Cerebrospinal fluid
CT: Computed tomography
EAN: European Academy of Neurology
ECDC: European Centre for Disease Prevention and Control
EEA: European Economic Area
ELISA: Enzyme-linked immunoassay
ER: Emergency room
EU: European Union
ICU: Intensive care unit
IgM: Immunoglobulin M
IRB: Institutional review board
NA: Not applicable
NSNS: Non-specific neurological symptoms
PCR: Polymerase chain reaction
SOP: Standard operating procedure
TBE: Tick-borne encephalitis

## Footnotes

1 The season is estimated to begin in March, with the highest frequency of TBE infections reported in June and July and risk of hospitalization peaking in August/September and decreasing by October [18].

2 The definition of endemicity was based on the number of designated risk areas within a federal state (at the time of conduct of the study), i.e., endemic (Bavaria, Baden-Wuerttemberg), middle endemic (Hesse, Saxony, Thuringia), low endemic (Saarland, Lower Saxony, Rhineland-Palatinate), and non-endemic (Berlin, Bremen, Hamburg, Schleswig–Holstein, Saxony-Anhalt, Mecklenburg-Western Pomerania, Brandenburg, North Rhine-Westphalia).
