# Supplementary material for: Evaluation of under-testing and under-diagnosis of tick-borne encephalitis in Germany
Source: BMC Infect Dis. 2023 Mar 7;23:139. doi: 10.1186/s12879-023-08101-6 (PMC9990549; doi:10.1186/s12879-023-08101-6)
Supplement: Supplementary file 1 — Additional file 1: TBE Under-Testing and Under-Diagnosis by Seasonality; the table shows frequencies and percentages of patients tested for TBE, not tested for TBE, and TBE positive for the total sample and symptom types stratified by admitted during TBE season and not admitted during TBE season. [file 12879_2023_8101_MOESM1_ESM.docx]

**Additional file 1. TBE Under-Testing and Under-Diagnosis by Seasonality**

| **Patients** | **N** | **TBE Tested,**  **n (%)** | **Not TBE Tested,**  **n (%)** | **TBE Positive,**  **n (%)** |
| --- | --- | --- | --- | --- |
| **Admitted during TBE season** | | | | |
| All patients | 749 | 521 (69.6) | 228 (30.4) | 147 (28.2) |
| Meningitis Only | 225 | 170 (75.6) | 55 (24.4) | 81 (47.6) |
| Encephalitis Only | 206 | 150 (72.8) | 56 (27.2) | 36 (24.0) |
| Myelitis Only | 144 | 93 (64.6) | 51 (35.4) | 9 (9.7) |
| Combination of Meningitis, Encephalitis, and/or Myelitis (≥2 of these) | 48 | 35 (72.9) | 13 (27.1) | 13 (37.1) |
| Non-specific Neurological Symptoms (exclusive) | 126 | 73 (57.9) | 53 (42.1) | 8 (11.0) |
| **Not admitted during the season** | | | | |
| All patients | 613 | 306 (49.9) | 307 (50.1) | 19 (6.2) |
| Meningitis Only | 176 | 79 (44.9) | 97 (55.1) | 11 (13.9) |
| Encephalitis Only | 143 | 79 (55.2) | 64 (44.8) | 5 (6.3) |
| Myelitis Only | 88 | 42 (47.7) | 46 (52.3) | 1 (2.4) |
| Combination of Meningitis, Encephalitis, and/or Myelitis (≥2 of these) | 19 | 10 (52.6) | 9 (47.4) | 1 (10.0) |
| Non-specific Neurological Symptoms (exclusive) | 187 | 96 (51.3) | 91 (48.7) | 1 (1.0) |

Note. The season is estimated to begin in March, with the highest frequency of TBE infections reported in June and July and risk of hospitalization peaking in August/September and decreasing by October. [18] Percentages values are row percentages. TBE: tick-borne encephalitis.

.
