# Supplementary material for: Evaluation of under-testing and under-diagnosis of tick-borne encephalitis in Germany
Source: BMC Infect Dis. 2023 Mar 7;23:139. doi: 10.1186/s12879-023-08101-6 (PMC9990549; doi:10.1186/s12879-023-08101-6)
Supplement: Supplementary file 2 — Additional file 2: Physicians Per Federal State: Observed Data Relative to the Population; the table shows the percentage of patients and the ratio of physicians per federal state observed in the current study to physicians per federal state in 2019 German population data for each of 16 federal states. [file 12879_2023_8101_MOESM2_ESM.docx]

**Additional file 2. Physicians Per Federal State: Observed Relative to the Population**

| **Federal state** | **Population Data (2019)** | | | | **TBE Data (Current Study)** | | | **Ratio of Physicians per Federal State TBE Data: Physicians per Federal State** |
| --- | --- | --- | --- | --- | --- | --- | --- | --- |
|  | **Population (n)** | **Patients (%)** | **Hospital Beds** | **Physicians per Federal State** | **Physicians per Federal State (n)** | **Patients (n)** | **Patients (%)** |  |
| North Rhine-Westphalia | 17947220 | 22% | 6.66 | 14.11 | 64 | 604 | 43% | 4.536 |
| Bavaria | 13124737 | 16% | 5.81 | 14.99 | 20 | 160 | 11% | 1.334 |
| Baden-Wuerttemberg | 11100394 | 13% | 5.01 | 13.80 | 13 | 109 | 8% | 0.942 |
| Lower Saxony | 7993608 | 10% | 5.24 | 13.98 | 4 | 29 | 2% | 0.286 |
| Hesse | 6288080 | 8% | 5.76 | 14.00 | 13 | 110 | 8% | 0.929 |
| Rhineland-Palatinate | 4093903 | 5% | 6.01 | 13.77 | 6 | 25 | 2% | 0.436 |
| Saxony | 4071971 | 5% | 6.44 | 15.01 | 8 | 61 | 4% | 0.533 |
| Berlin | 3669491 | 4% | 5.61 | 17.62 | 26 | 206 | 15% | 1.476 |
| Schleswig-Holstein | 2903773 | 3% | 5.44 | 14.75 | 1 | 10 | 1% | 0.068 |
| Brandenburg | 2521893 | 3% | 6.13 | 13.60 | 1 | 10 | 1% | 0.074 |
| Saxony-Anhalt | 2194782 | 3% | 6.98 | 14.56 | 0 | 0 | 0% | 0.000 |
| Thuringia | 2133378 | 3% | 7.48 | 15.11 | 1 | 10 | 1% | 0.066 |
| Hamburg | 1847253 | 2% | 6.89 | 18.02 | 8 | 66 | 5% | 0.444 |
| Mecklenburg-Western Pomerania | 1608138 | 2% | 6.34 | 15.33 | 0 | 0 | 0% | 0.000 |
| Saarland | 986887 | 1% | 6.84 | 15.68 | 0 | 0 | 0% | 0.000 |
| Bremen | 681202 | 1% | 7.39 | 18.45 | 0 | 0 | 0% | 0.000 |
| **Total** | 83166710 | -- | -- | -- | -- | 1400 | -- | -- |

Note. TBE: tick-borne encephalitis.
